# Use of trained scent dogs for detection of COVID-19 and evidence of cost-saving

**DOI:** 10.3389/fmed.2022.1006315

**Published:** 2022-12-01

**Authors:** Leon Mutesa, Gashegu Misbah, Eric Remera, Hans Ebbers, Esther Schalke, Patrick Tuyisenge, Reuben Sindayiheba, Clement Igiraneza, Jeanine Uwimana, Diane Mbabazi, Epimaque Kayonga, Michel Twagiramungu, Denyse Mugwaneza, Leandre Ishema, Yvan Butera, Clarisse Musanabaganwa, Edson Rwagasore, Friederike Twele, Sebastian Meller, Albert Tuyishime, Robert Rutayisire, Marilyn Milumbu Murindahabi, Lindsay A. Wilson, Noella Bigirimana, Holger A. Volk, Vedaste Ndahindwa, Benoit Kayijuka, Edward J. Mills, Claude Mambo Muvunyi, Sabin Nsanzimana

**Affiliations:** ^1^Center for Human Genetics, Inc., College of Medicine and Health Sciences, University of Rwanda, Kigali, Rwanda; ^2^Rwanda National Joint Task Force COVID-19, Kigali, Rwanda; ^3^Kynoscience UG, Praxis und Wissenschaft, Hörstel, Germany; ^4^K9 Department, Rwanda National Police, Kigali, Rwanda; ^5^Department of Small Animal Medicine and Surgery, University of Veterinary Medicine Hannover, Hannover, Germany; ^6^School of Population and Public Health, University of British Columbia, Vancouver, BC, Canada; ^7^Department of Health Research Methods, Evidence and Impact, McMaster University, Hamilton, ON, Canada

**Keywords:** COVID-19, SARS-CoV-2, volatile organic compounds (VOCs), scent dogs, RT-PCR, cost-saving

## Abstract

**Background:**

One of the lessons learned from the coronavirus disease 2019 (COVID-19) pandemic is the importance of early, flexible, and rapidly deployable disease detection methods. Currently, diagnosis of COVID-19 requires the collection of oro/nasopharyngal swabs, nasal turbinate, anterior nares and saliva but as the pandemic continues, disease detection methods that can identify infected individuals earlier and more quickly will be crucial for slowing the spread of the virus. Previous studies have indicated that dogs can be trained to identify volatile organic compounds (VOCs) produced during respiratory infections. We sought to determine whether this approach could be applied for detection of COVID-19 in Rwanda and measured its cost-saving.

**Methods:**

Over a period of 5 months, four dogs were trained to detect VOCs in sweat samples collected from human subjects confirmed positive or negative for COVID-19 by reverse transcription polymerase chain reaction (RT-PCR) testing. Dogs were trained using a detection dog training system (DDTS) and *in vivo* diagnosis. Samples were collected from 5,253 participants using a cotton pad swiped in the underarm to collect sweat samples. Statistical analysis was conducted using R statistical software.

**Findings:**

From August to September 2021 during the Delta wave, the sensitivity of the dogs’ COVID-19 detection ranged from 75.0 to 89.9% for the lowest- and highest-performing dogs, respectively. Specificity ranged from 96.1 to 98.4%, respectively. In the second phase coinciding with the Omicron wave (January–March 2022), the sensitivity decreased substantially from 36.6 to 41.5%, while specificity remained above 95% for all four dogs. The sensitivity and specificity by any positive sample detected by at least one dog was 83.9, 95% CI: 75.8–90.2 and 94.9%; 95% CI: 93.9–95.8, respectively. The use of scent detection dogs was also found to be cost-saving compared to antigen rapid diagnostic tests, based on a marginal cost of approximately $14,000 USD for testing of the 5,253 samples which makes 2.67 USD per sample. Testing turnaround time was also faster with the scent detection dogs, at 3 h compared to 11 h with routine diagnostic testing.

**Conclusion:**

The findings from this study indicate that trained dogs can accurately identify respiratory secretion samples from asymptomatic and symptomatic COVID-19 patients timely and cost-effectively. Our findings recommend further uptake of this approach for COVID-19 detection.

## Introduction

Since its recognition as a public health emergency of international concern in January 2020, the coronavirus disease 2019 (COVID-19) has spread around the world, and was declared a pandemic by the World Health Organization (WHO) in March, 2020 (1). Effective management of infectious diseases depends on reliable and timely diagnosis (2) and in the case of COVID-19, the gold standard diagnostic test is the Reverse Transcription-Polymerase Chain Reaction (RT-PCR) test using oro/nasopharyngeal swabs or other upper respiratory tract specimens. Unfortunately, this method of testing is not widely available in low- and middle-income countries (LMICs) due to the lack of reagents’ supply and low testing capacity (3). Furthermore, RT-PCR tests can be time-consuming to process, and can produce false positive or negative results (3). These limitations have led to significant challenges in LMICs. In early 2020, the Government of Rwanda built on existing RT-PCR testing capabilities acquired during the Ebola Virus Disease (EVD) epidemic to improve early detection of COVID-19 (Rwanda COVID-19 Intra Action Review, 2020). The ability to detect COVID-19 (either using RT-PCR or rapid antigen tests) was rapidly extended to all healthcare facilities in the country. However, there were challenges due to the complexity of RT-PCR testing, and although new innovative testing strategies were developed, these approaches still required extensive laboratory equipment and trained laboratory experts (4). These challenges resulted in delays of both case detection and management. While the recent introduction of rapid antigen tests has significantly reduced the turnaround, time needed to provide patients with results, there is still a need for faster and easier ways of detecting COVID-19 to enable appropriate and cost effective COVID-19 test.

One approach to the rapid detection of COVID-19 is through the use of medical scent detection dogs, which can rapidly detect volatile organic compounds (VOCs) associated with coronavirus with a high degree of specificity, sensitivity, and accuracy for a large number of individuals (5, 6). Evidence of dogs’ efficacy in detecting medical conditions and diseases (either communicable or non-communicable) has been reported in studies conducted in Germany and UK (7, 8). In Germany for example, a study conducted with eight detection dogs on 1,012 randomized samples resulted in an overall detection rate of 94%, while sensitivity and specificity rates were 82.63 and 96.35%, respectively (9). Several studies have shown the ability of medical scent detection dogs to identify samples from SARS-CoV-2 infected individuals with high accuracy, highlighting the role such dogs could play in the management of a pandemic (10–13). Previous research showed that different body fluids, such as saliva, sweat and urine and other sample types like worn face masks are suitable for detection, which suggests that there is a general SARS-CoV-2 infection associated odor that dogs can be trained on (13, 14). In addition, our group demonstrated that such dogs were able to differentiate SARS-CoV-2 infection from other acute viral respiratory tract infections (7). However, most of the current data were generated in laboratory settings, rather than in a real-world scenario.

Our study sought to test the concept of using dogs to reliably differentiate between samples from patients infected with COVID-19 and non-infected controls in Rwanda. To our knowledge, this is the first study of its kind to be conducted in a LMIC.

## Materials and methods

### Study design and setting

This study was a cross-sectional design to assess the validity of the scent dog test for COVID-19 using sweat samples from both symptomatic and asymptomatic patients. Between March and July 2021, we performed trainings of dogs and handlers in regard to the sensitivity and specificity compared to RT-PCR gold standard’s results, followed by a pilot using 61 known samples. These sweat samples and oro-nasal pharyngeal swabs were collected from symptomatic and asymptomatic individuals as described below. In this pilot phase, dogs learned to identify COVID-19 sweat samples directly by smelling the human body odors present in a cotton pad that participants swiped in their armpit. After this pilot phase, in August 2021 we initiated the first validation phase where four dogs were continuously trained to detect COVID-19 in sweat samples collected from both symptomatic and asymptomatic individuals admitted at King Faisal Hospital and the University Teaching Hospital of Kigali (CHUK), respectively. In addition, we also collected samples from the participants recruited across the country in high spot areas from the City of Kigali, Western, Southern, Northern, and Eastern provinces of Rwanda. Samples were also collected from markets, bars, restaurant, and churches during random drive through national outreach COVID-19 testing campaigns. This phase coincided with the wave associated with the surge of Delta variant which took place between July and mid-December 2021 in Rwanda.

In the second validation phase corresponding to the wave of Omicron variant which started late December 2021, we continued to collect and process the same samples until March. There was no incentive involved in the recruitment and sample collection process. All tests were performed free of charge as part of national response to COVID-19 in the interest of public health.

### Sample size

In total, 5,253 sweat samples (in addition to 61 samples collected during the pilot) were collected from symptomatic, asymptomatic and non-infected individuals for COVID-19 patients aged 18 years and above from August 2021 to March 2022 covering two periods of Delta and Omicron variants’ waves.

### Specimen collection

Two types of samples were collected from consented both symptomatic patients and non-infected individuals upon their arrival at the hospital or site of sample collection. The first sample type was an oro-pharyngeal swab collected from the tonsils and posterior pharynx wall. Swab heads were immersed in 3 ml Viral Transport Medium (VTM), following manufacturer’s guidelines, and then sent directly to the National Reference Laboratory (NRL/RBC) for RT-PCR testing. The second sample type was a self-collected sweat sample from all symptomatic and asymptomatic patients. Each patient was briefed on proper sample self-collection, which comprised of swiping a cotton pad (Wattenschijfjes Disque à Démaquiller, Everyday) in both armpits for at least 5 min and placing it into a glass jar. Samples were stored in the laboratory between 4–8°C until the time of testing, and at −80°C for long term bio-banking. In addition, we also collected saliva samples for bio-banking for further studies.

### Reverse transcription polymerase chain reaction testing

Reverse transcription polymerase chain reaction was considered the gold standard test against which to compare the scent detection dogs’ performance. All dog handlers were fully equipped with proper personal protective equipment (PPE) every time they were handling dogs or/and samples. Oropharyngeal RNA samples were extracted with a DAAN RNA/DNA Purification Kit (8). A total of 5 μl of extracted RNA were added to 20 μl of a master mix to make a solution of 25 μl, as per manufacturer’s guidelines. The RT-PCR test for detection of SARS-CoV-2 was done using 2019-nCoV RNA RT-PCR kit targeting two genes [orf1ab1ab known as open reading frame and nucleocapsid protein (*N*)] as described by manufacturer (DAAN Gene Co., Ltd., Of Sun Yat-sen University, 19, Xiangshan Road, Guangzhou Hi-Tech Industrial Development Zone, China). The solution was run on the Bio-Rad CFX96 thermocycler at 50°C for 15 min for reverse transcription, denatured at 95°C for 15 min, followed by 45 PCR cycles at 94°C for 15 s and 55°C for 45 s. The average turnaround time for RT-PCR was 21/2 h. A cycle threshold value (C*t*) of more than or equal to 37 indicated a negative test result. Positive controls for the reaction showed amplification as determined by curves for FAM and VIC detection channels (4).

### Sniffer dogs’ characteristics

This study was conducted in collaboration with the canine department of the Rwanda National Police at Kigali International Airport. The dogs were supplied by Police Dogs Centre Holland BV, RJ Sint-Oedenrode, The Netherlands. They were selected according to features such as age, breed, and sex. The dogs’ characteristics are displayed in Table 1.

**TABLE 1:** Dogs’ characteristics.

Name	Age (year)	Sex	Breed
Dog 1	2	Male	Labrador
Dog 2	2	Male	Labrador
Dog 3	2	Male	Malinois
Dog 4	2	Female	Malinois

### Detection dog training system

**FIGURE 1:**
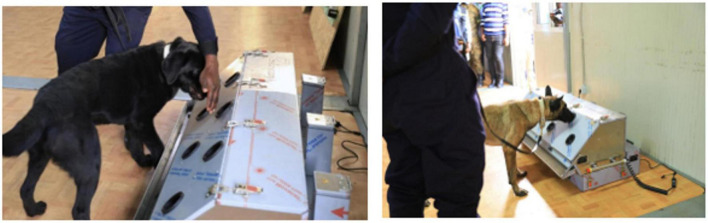
The Detection Dog Training System (DDTS, Kynoscience UG, Germany) used for training dogs. This machine system has seven sniffing holes attached to tubes where scent dogs detect COVID-19 samples.

The Detection Dog Training System (DDTS, Kynoscience UG, Germany) was used for training dogs. The system is composed of seven “sniffing holes” attached to tubes (Figure 1). Behind each hole there is a tube leading to a metal container. Each metal container is covered with a grid, which allows the odor to escape and reach the sniffing hole. Each tube L-shaped order to prevent physical contact with the samples and to avoid any visual cues that may impact results.

### Scent dog detection facility set up and use of olfactory cones

**FIGURE 2:**
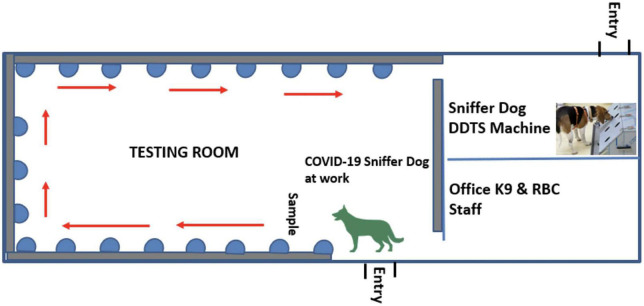
A scent dog detection facility constructed at the Kigali International Airport comprising of three rooms including the testing room, the DDTS room, and a staff room.

The scent dog detection facility was set-up at Kigali International Airport with objective to scale-up this testing strategy in collaboration with Canine brigade of Rwanda National Police and Rwanda Airports Company and for strengthening infection prevention and control (IPC) measures against COVID-19 in the country with limited cost. This facility was made up of three rooms including the testing room, the DDTS room, and a staff room (Figure 2).

**FIGURE 3:**
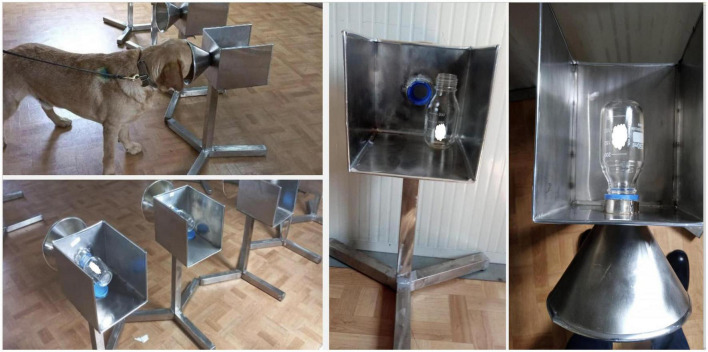
Locally manufactured olfaction cones used by dogs for detection of COVID-19 in sweat samples collected from individuals on a cotton pad carried in a glass container. The containers are covered with grids, which allowed the odor to escape and reach the sniffing hole. Each tube extension is identical and shaped in a way that it prevents dogs from physical contact with the samples.

As the DDTS machine has a limited throughput related to logistics, custom olfactory cone products were developed for the actual specimen testing and for easy scale-up locally. These olfactory cones were locally made from a funnel to be used by the dogs during the detection of VOCs. The funnels were attached to a bottle containing a cotton pad used to collect sweat samples (Figure 3).

### Training of handlers and scent dogs for detection of COVID-19

Dogs’ handlers received a pilot training in basic commands, dogs’ learning behavior, and different rewarding methods. The four dogs were first trained for detection of COVID-19 using DDTS. The dogs were introduced to the sweat samples of patients with COVID-19 and healthy controls so that they become familiar with these secretions. Sweat samples stored in appropriate storage temperature as described in specimen collection method. After being collected samples were transported every day from the National Reference Laboratory to the training site at Kigali International Airport. The samples were then placed in the olfactory cones and each dog smelled the secretion in each cone in order to learn how to distinguish positive from negative samples. Each dog smelled each sample for around 1 s and then moved to the next one. When the dog indicated a positive sample, the dog stopped at the olfaction cone for 3–4 s. The dog indication behavior attracted attention of the handlers, and the dog was rewarded and then continued to next cones.

After intensive training, each dog could smell an average of 50 samples within 3 min. After each day’s training, samples were re-stored in Kigali International Airport Molecular Laboratory.

### Safety measures

Before starting the study, the dogs’ handlers were tested for COVID-19 using RT-PCR testing. The handlers were also familiar about COVID-19 symptoms and how to respond to a potential exposure. They were then re-tested regularly every 2 weeks over the course of the pilot study. COVID-19 prevention measures were taken to prevent infection throughout the study, including the use of PPE (i.e., face masks, face shields, and lab coats). All samples were transported in accordance with recommended procedures. The dogs were kept in standard crates in accordance with ethical guidelines, and were fed high-quality dog food throughout the study by veterinary doctors.

### Statistical analysis

We disaggregated our analysis into two time periods that correspond with waves of differing dominant COVID-19 variants in the country during the study period. The first period was from August to September 2021, when the Delta variant was dominant, while the second period was from January to March 2022, when the Omicron variant was dominant. The period from October to December 2021 was removed from the analysis as there was no positive case identified by the PCR test even if scent dogs continued testing. We combined results from all dogs to generate a new binary variable (1: positive with at least one dog and 0 for negative to all dogs). This categorization was based on probable impact of variant to the performance of scent dogs. Sensitivity, specificity, positive predictive value, and negative predictive value as well as the Receiver Operating Characteristic (ROC) were calculated in comparison with the RT-PCR results, considered as gold standard. In addition, the agreement level and a Kappa coefficient (k) was calculated to measure the level of agreement between scent detection dogs and RT-PCR testing.

## Results

In our analysis of detection dogs’ diagnostic performance with sweat samples, a total of 5,253 sweat samples were collected from major hospitals, treatment centers, markets, churches, and other hot spot areas across the country during the peak of the Delta and Omicron variants. Overall, 4.0% (123/3,071) of individuals tested positive for COVID-19 using RT-PCR. Results show a high positive yield of 12.4% (84/678) in period-1 (August to September 2021) and 1.63% (23/2.393) in period-2 (January to March 2022) (*P* < 0.05). Similarly, the positive yield using sniffer dogs ranged from 11.8 to 13.7% in period 1 and from 2.4 to 3.9% in period-2. The Kappa coefficient varied from 0,7 to 0,9 in the period-1 indicating a substantial agreement. However, results showed that the kappa coefficient was reduced to 0.3 and 0.2 in the period-2, showing a fair agreement (Table 2).

**TABLE 2:** Number of sniffed sweat samples per dog and level of agreement with RT-PCR.

Dog	Period	Number of sniffed sweat samples	Tested positive	Agreement with RT-PCR test %	Kappa (k)*
				
			*n* (%)	(95% CI)	
Dog 1	Total	3,071	144 (4.7%)	98.0 (97.3; 98.8)	0.6
	August–September 2021	678	80 (11.8%)	96.5 (95.5; 97.5)	0.8
	January–March 2022	2,393	64 (2.7%)	96.9 (95.9; 97.8)	0.3
Dog 2	Total	3,057	166 (5.4%)	97.9 (97.1; 98.6)	0.6
	August–September 2021	664	89 (13.4%)	96.8 (95.8; 97.8)	0.9
	January–March 2022	2,393	77 (3.2%)	96.5 (95.6; 97.4)	0.3
Dog 3	Total	2,842	144 (5.1%)	97.9 (97.2; 98.7)	0.6
	August–September 2021	678	93 (13.7%)	94.3 (93.3; 95.3)	0.7
	January–March 2022	2,164	51 (2.4%)	97.3 (96.3; 98.2)	0.3
Dog 4	Total	2,497	153 (6.1%)	96.5 (95.6; 97.3)	0.6
	August–September 2021	664	80 (12.1%)	94.4 (93.4; 95.4)	0.7
	January–March 2022	1,833	73 (3.9%)	95.6 (94.6; 96.5)	0.2

*Kappa coefficient (k) helps to measure the level of agreement produced during the detection of SARS-CoV-2 between scent dogs and RT-PCR.

From August to September 2021 while we were in the period of Delta wave, the sensitivity of the dogs’ COVID-19 detection ranged from 75.0 to 89.9% for the lowest- and highest-performing dogs, respectively. Specificity ranged from 96.1 to 98.4%, respectively. In the second period coinciding with the Omicron wave (January–March 2022), the sensitivity decreased substantially ranging from 36.6 to 41.5%, while specificity remained above 95% for all four dogs (Table 3). The sensitivity and specificity by any positive detected by at least one dog were 83.9, 95% CI: 75.8–90.2 and 94.9%; 95% CI: 93.9–95.8, respectively.

**TABLE 3:** The detection dogs’ performance.

			Period 1		Period 2	
				
	Overall		(August–September 2021)		(Jan–March 2022)	
			
	Percent	95% CI	Percent	95% CI	Percent	95% CI
**Dog 1**						
Sensitivity	68.5	(59.7–76.3)	83.1	(73.7–90.2)	36.6	(22.1–53.1)
Specificity	98.0	(97.4–98.4)	98.4	(97.1–99.2)	97.9	(97.2–98.4)
ROC area	83.2	(79.2–87.2)	90.8	(87.0–95.0)	67.2	(59.8–74.7)
Positive predictive value	59.3	(51.0–67.3)	88.1	(79.2–94.1)	21.2	(11.1–34.7)
Negative predictive value	98.6	(98.1–99.0)	97.6	(96.1–98.7)	99.1	(98.6–99.3)
**Dog 2**						
Sensitivity	70.6	(61.5–78.6)	82	(72.5–89.4)	36.7	(19.9–56.1)
Specificity	97.7	(97.0–98.2)	96.1	(94.3–97.5)	98.1	(97.4–98.6)
ROC area	84.1	(80.0–88.2)	89.1	(85.0–93.0)	67.4	(58.6–76.2)
Positive predictive value	56.4	(48.0–64.5)	75.3	(65.5–83.5)	41.9	(29.1–55.7)
Negative predictive value	98.7	(98.2–99.1)	97.4	(95.8–98.5)	97.6	(96.9–98.2)
**Dog 3**						
Sensitivity	74.6	(66.2–81.8)	89.9	(81.7–95.3)	41.5	(26.3–57.9)
Specificity	97.4	(96.7–97.9)	97.4	(95.7–98.5)	97.4	(96.6–98.0)
ROC area	86.0	(82.2–89.8)	93.6	(90.0–97.0)	69.0	(61.8–77.0)
Positive predictive value	55.1	(47.4–62.6)	83.0	(74.4–90.2)	21.3	(12.9–31.8)
Negative predictive value	98.9	(98.4–99.2)	98.5	(97.2–99.3)	99.0	(98.5–99.3)
**Dog 4**						
Sensitivity	64.1	(55.1–72.3)	75.0	(64.6–83.6)	40.0	(24.9–56.7)
Specificity	96.9	(96.1–97.5)	97.2	(95.6–98.4)	96.7	(95.8–97.5)
ROC area	80.5	(76.0–85.0)	86.1	(82.0–91.0)	68.4	(60.7–76.1)
Positive predictive value	51.9	(43.8–59.9)	79.5	(69.2–87.6)	21.3	(12.7–32.3)
Negative predictive value	98.1	(97.4–98.6)	96.4	(94.6–97.7)	98.7	(98.0–99.1)
**At least one dog**						
Sensitivity	83.9	(75.8–90.2)	97.6	(91.6–99.7)	44.8	(26.4–64.3)
Specificity	94.9	(93.9–95.8)	92.6	(90.1–94.6)	95.7	(94.6–96.7)
ROC area	90.0	(86.0–92.9)	95.1	(93.1–97.1)	70.3	(61.1–79.5)
Positive predictive value	46.3	(39.3–53.4)	65.9	(56.8–74.2)	16.3	(8.95–26.2)
Negative predictive value	99.1	(98.6–99.5)	99.6	(98.6–100.0)	99.0	(98.3–99.4)

**FIGURE 4:**
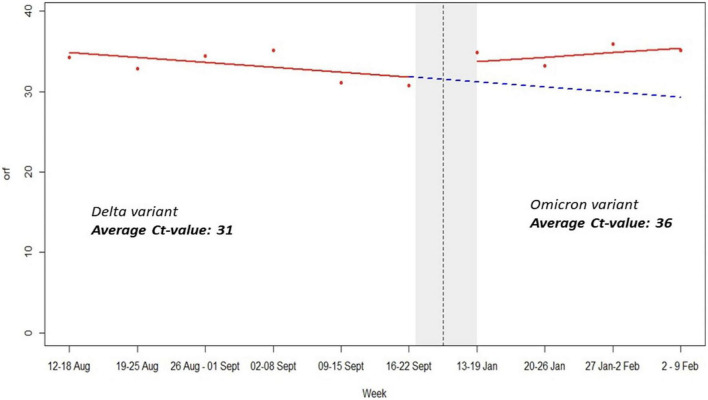
Impact of variants C*t*-values on dogs’ performance.

The period of delta variant was characterized by low orf1ab and *N* genes C*t*-values, severe symptoms, many deaths and high viral load while omicron variant period was marked by high C*t*-values, mild symptoms, low viral load, and very few deaths (Figure 4). This is scientific evidence regarding impact of SARS-CoV-2 variants vs. sniffer dogs’ performance. It is worth noting that mean average C*t*-values for RT-PCR SARS-CoV-2 detection rate was 31 and 36 during Delta and Omicron waves, respectively (Figure 4).

### Cost minimization analysis and turnaround time

**FIGURE 5:**
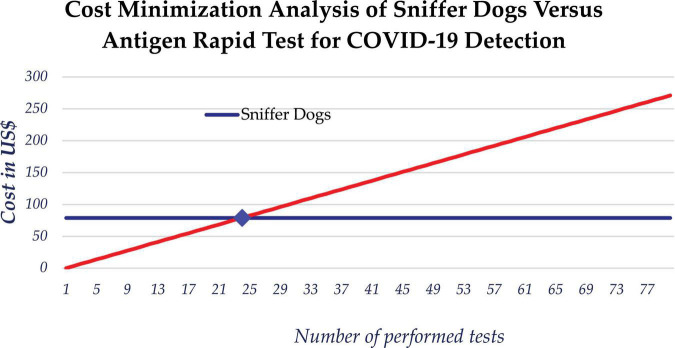
Cost minimization analysis between use of scent dogs and rapid antigen tests for detection of COVID-19.

We also considered the cost effectiveness of using scent dogs for detection of COVID-19 compared to rapid antigen test. While the cost of mass testing for COVID-19 using dogs is relatively constant over the number of screened persons, the cost of using rapid antigen tests increases with the number of tests performed. The estimated daily average cost of scent detection dogs was $79 USD, which is approximately equivalent to the cost of 24 rapid tests. The use of scent detection dogs was found to be cost-saving compared to Antigen rapid diagnostic tests, based on a marginal cost of approximately $14,000 USD for testing of the 5,253 samples which makes 2.67 USD per sample. When testing more than 24 samples, the use of dogs could minimize the cost of testing (Figure 5).

For estimating TAT, we calculated unit time in minute for testing using both RDT and scent detection dog (Table 4). Different variables including testing preparation, sample collection, sample transportation, sample processing, results and recording times have been considered for demonstrating TAT corresponding to each testing method. Overall results showed that the use of scent detection dog for testing one sample was 6.7 min per sample, while the use of RDT had an average TAT of 12.13 min per sample.

**TABLE 4:** Turnaround time estimation for both scent detection dog and RDT per one sample (unit time = minute).

Process	Time for Ag-RDT	Time for scent dog
Preparation of materials for testing	2.00	2.00
Sample coding and registration	2.00	2.00
Sample collection	0.17	0.20
Sample transportation	0.00	1.00
Sample processing and results reading	7.33	0.17
Result recording into the information system	1.00	1.00
Total time	12.50	6.37

## Discussion

This study demonstrated that the use of trained dogs for the detection of COVID-19 is a viable mass screening diagnostic approach with evidence of cost-savings. The use of scent detection dogs to detect diseases is not new in medical history. Different studies have demonstrated the ability of dogs to detect specific odors from individuals with certain types of diseases (7, 10, 11). Other studies examining the capacity of scent detection dogs to detect COVID-19 have reported results ranging from 76 to 100% success rates after 1 week of training (2, 9). Furthermore, the use of scent detection dogs represents a faster, cheaper way of disease detection that requires less technology, minimal training of operators and avoids direct contact between clients and sample collectors, thereby potentially limiting disease spread (12, 13).

In our study of 5,253 samples, detection dogs were able to distinguish infected COVID-19 patients’ using armpit sweat samples with good sensitivity and excellent specificity. Our findings indicate that scent detection dogs may contribute effectively to the safe resumption of activities while also helping to keep COVID-19 infections under control. This is especially noteworthy in low-resource settings where testing resources and capacity may be limited. The variation of scent dogs’ sensitivity and specificity observed during the two study periods is likely explained by the impact of the Delta and Omicron variants. Based on epidemiological data and genomic dynamics of SARS-CoV-2 in Rwanda, the peak of the Delta wave was observed in August 2021 while the peak of the Omicron wave occurred in January 2022 (13). In addition, other factors such as immunity status post-natural infection or vaccination as well as time of diagnostics and sample collection may explain the C*t*-values’ variations during both waves. Indeed, during the Delta variant wave, the orf1ab and N-genes’ C*t*-values were low while the scent dogs’ sensitivity and specificity were 98 and 82.1%, respectively. The period of delta variant wave was characterized by low orf1ab and N genes C*t*-values, severe symptoms, many deaths and high viral load, while the omicron variant wave was marked by high C*t*-values, mild symptoms, low viral load and very few deaths (Figure 4). It is important to mention that during the Delta period, the majority of patients who tested positive were symptomatic, likely manifesting in a higher viral load compared to patients who tested positive during the Omicron wave and were often asymptomatic, potentially impacting the detection ability of the dogs. This evidence has been demonstrated by previous studies that have indicated that low C*t*-values are inversely proportional to viral load in COVID-19 patients (9).

Our study findings also indicate that the use of scent detection dogs is cost-effective. Furthermore, scent dogs require limited resources to deploy, and significantly reduce the turnaround time needed to provide results to patients compared to Polymerase Chain Reaction and Antigen Rapid Tests. The cost of mass testing for COVID-19 using scent detection dogs is relatively constant regardless the number of screened persons. Using these dogs during mass testing for COVID-19 would be very beneficial by limiting the cost and responding to the challenge of procurement and distribution of rapid antigen test.

There are some strengths and limitations to this study. The overall strengths include our large sample size obtained from a diverse population across the country, and our inclusion of both symptomatic and asymptomatic patients. Our study also represents the first of its kind to be conducted in a low-income setting, and demonstrates the feasibility of this approach across socio-economic contexts. A limitation of our study is the relatively small number of SARS-CoV-2 positive samples included in our sample due to the successful containment of COVID-19 in Rwanda at the time of our data collection and analysis.

## Conclusion

In conclusion, it should be noted that although dogs hold potential as real-time detectors of VOCs, they require intense training and meticulous selection of the best performing dogs before deployment. Interestingly, the variation in dogs’ performance could be affected by emerging COVID-19 variants and thus regular refresher training courses are highly recommended for better infection control. Furthermore, as the use of scent detection dogs expands, it is important to take precautions to avoid any risk of contagion while dogs interact with infected human samples. In our study, we designed custom samples holders with double protection systems to protect the dogs from being infected. As the world prepares for future pandemics, trained dogs may offer an important addition to existing diagnostic tools. Subsequent studies could assess the capability of the trained dogs to detect asymptomatic SARS- CoV-2 infection, and then the deployment of dogs in the field and at entry points to support ongoing efforts and COVID-19 response strategies.

## Data availability statement

The original contributions presented in this study are included in the article. Further inquiries can be directed to the corresponding author.

## Ethics statement

This study partially involving human participants was reviewed and approved by the Rwanda National Ethics Committee; RNEC/2020-No. 856/RNEC/2021 (FWA assurance no. 00001973-IRB00001497 of IORG0001100). For the use of animals, we did not need an ethics as these were service dogs and this study was approved in accordance with local guidelines. In addition, this study was implemented in the framework of emergency National response to COVID-19 under the coordination of national joint COVID-19 response committee that oversees all COVID response interventions thus did not need a formal ethics approval for use of these service dogs.

## Author contributions

LM and SN: conceptualization, funding acquisition, and supervision LM, GM, ErR, HE, ES, PT, RS, CI, JU, DiM, EK, MT, YB, CM, EdR, FT, SM, AT, RR, MM, VN, BK, CMM, LW, and SN: investigation, methodology, validation, and data curation. LM, GM, ErR, PT, RS, CI, JU, VN, BK, and SN: writing—original draft. LM, GM, EdR, PT, RS, CI, JU, NB, HV, VN, CM, BK, LW, EM, CMM, and SN: writing—review and editing. LM, GM, BK, and SN: project administration. All authors have read and agreed to the published version of the manuscript.
